# The Impact of Global Transcriptional Regulation on Bacterial Gene Order

**DOI:** 10.1016/j.isci.2020.101029

**Published:** 2020-04-02

**Authors:** Pablo Yubero, Juan F. Poyatos

**Affiliations:** 1 Logic of Genomic Systems Laboratory, CNB - CSIC, Madrid 28049, Spain

**Keywords:** Microbiology, Microbial Genetics, Mathematical Biosciences

## Abstract

Bacterial gene expression depends on the allocation of limited transcriptional resources provided a particular growth rate and growth condition. Early studies in a few genes suggested this global regulation to generate a unifying hyperbolic expression pattern. Here, we developed a large-scale method that generalizes these experiments to quantify the response to growth of over 700 genes that *a priori* do not exhibit any specific control. We distinguish a core subset following a promoter-specific hyperbolic response. Within this group, we sort genes with regard to their responsiveness to the global regulatory program to show that those with a particularly sensitive linear response are located near the origin of replication. We then find evidence that this genomic architecture is biologically significant by examining position conservation of *E*. *coli* genes in 100 bacteria. The response to the transcriptional resources of the cell results in an additional feature contributing to bacterial genome organization.

## Highlights

• Cell physiology determines a global transcriptional regulatory program
• Constitutive genes show a differential response to this global regulation
• The most responsive constitutive genes are located near the origin of replication
• Global transcriptional regulation acts as a gene position conservation force

## Graphical Abstract

**Figure:**
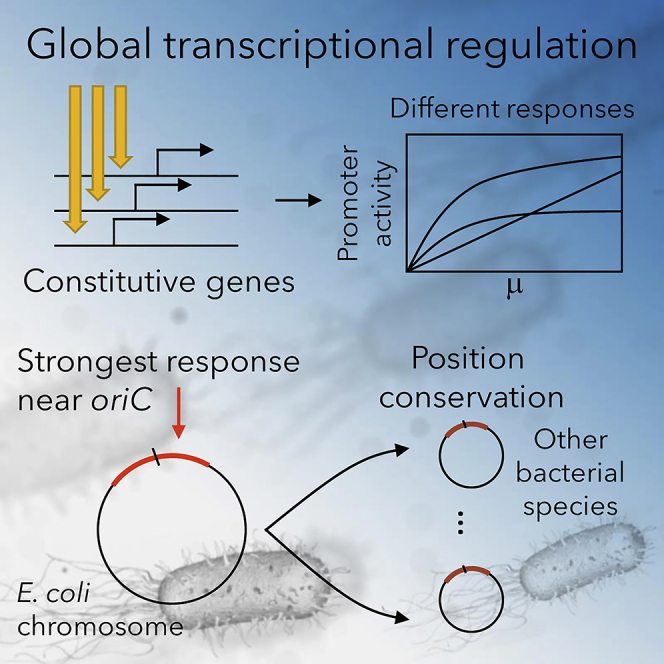

## Introduction

Transcription regulation is one of the fundamental mechanisms by which bacteria adapt gene expression to changing environmental conditions. Apart from the specific action mediated by transcription factors (TFs), expression is modulated by a global regulatory program determined by the physiological condition of the cell. Initial studies correlated this condition to the availability of core constituents of the expression machinery: free RNA polymerase, tRNAs, ribosomes, etc. (Kjeldgaard et al., 1958, Schaechter et al., 1958), but many other interacting components can play a role such as the cell volume, or the alarmone (p)ppGpp (Kubitschek, 1974, Liang et al., 1999, Traxler et al., 2008). These works also provided an effective protocol to describe the influence of all these elements: the dependence on physiology was linked to growth rate at exponential phase independent of the particular nutrients fixing that rate. Therefore the examination of the global program reduced to the quantification of expression response to changes in growth rate and growing conditions.

The fact that global physiology complements specific regulation matters in many aspects. Indeed, growth rate dependencies can interfere directly with genetic circuits and change their operation, for example, by shifting the bistability regime of a switch or allowing for different antibiotic resistance strategies (Deris et al., 2013, Klumpp et al., 2009). Costs of synthetic genetic circuits on cell physiology and the consequences of the latter on the function of the circuits made the subject also relevant in applied areas, e.g., Synthetic Biology (Scott et al., 2010). Beyond “simple” genetic circuits, the interplay of global regulation and cell resource allocation can modify many essential features at the system level (Klumpp and Hwa, 2008, Peebo et al., 2015). In fact, mechanistic approaches revealed that the global regulatory program contributes to determining fundamental trade-offs involving the finiteness of the cellular size, energy, and ribosomal fraction (Weiße et al., 2015).

To examine the activity of this program, the choice of constitutive genes as the primary model is clear: promoters of these genes lack any interaction with specific DNA-binding TFs, and thus, they are *a priori* constantly available for transcription initiation. Therefore, constitutive genes are subject only to physiological regulation. An alternative approach is to mutate the TF-binding sites of non-constitutive genes to assess the separate (mutant) and combined (wild-type) effect of global and specific regulation. Studies applying these approximations included, however, only a few genes (Berthoumieux et al., 2013, Gerosa et al., 2013, Kochanowski et al., 2017), and thus we lack a large-scale evaluation of global transcriptional regulation.

Beyond its evaluation, it is also intriguing to examine to what degree global regulation could impact bacterial genomic organization, as it is the case for specific regulation (Camas and Poyatos, 2008). One of the factors contributing to this regulation is copy number as gene dosage depends on the growth rate and on the distance to the origin of replication *oriC* of the chromosome. This is due to the overlap of multiple replication rounds at fast growth rates (multifork replication). Indeed, the position and copy number of ribosomal genes in *Escherichia coli* are tuned to maintain fast growth rates (Gyorfy et al., 2015). We could, nevertheless, ask if the global transcriptional regulation excluding the copy number affects genomic architecture. At least two scenarios can be postulated. In one scenario, promoters that are intrinsically sensitive to the global transcriptional regulation, i.e., excluding copy number contribution, are located far from *oriC* to compensate for the small, almost negligible, increase in copy number at large growth rates. In the second scenario, those promoters are located near *oriC* to further enhance their activity with growth rate. In the first situation, the influence of the global transcriptional program is compensated along the chromosome, whereas in the second, copy number strengthens the dependence between expression and growth rate. Either solution would reveal design principles of genome architecture.

In this work, we introduce a procedure that enables us to first examine at a large scale the response of ∼700 genes with no known explicit regulation by TFs to the separate and combined effect of the global regulation program and the chromosomal copy number variations due to multifork replication in *E*. *coli*. To this end, we develop a method that uses experimental time series of growth rate and promoter activity of a fluorescent reporter library (Zaslaver et al., 2009, Zaslaver et al., 2006), which has been proved to be one of the best tools to study *in vivo* gene expression at large scale. This allowed us to recognize a core set of strictly constitutive genes presenting a promoter-dependent hyperbolic response. For these genes, we quantify the most sensitive to the global program and observe that they are significantly located near the origin of replication. This presents the proximity to *oriC* as an important feature to enhance the control of expression and suggests that the location of these genes could be particularly conserved in species in which this control is desirable, e.g., those experiencing faster or more variable growth rates. We examine evidence in this respect with the analysis of the correlation between position conservation of the corresponding *E*. *coli* genes in 100 bacterial species and the number of replication rounds, maximal growth rate, and environmental variability of the species' habitat.

## Results

### Quantifying Chromosomal Promoter Activity at a Large Scale

To quantify the promoter activity of chromosomal genes (PA_*chr*_) we developed a method that makes use of promoter activity measurements obtained with low-copy plasmids (PA_*pl*_). This is of particular interest as the availability of a fluorescent library in *E*. *coli* (Zaslaver et al., 2006) could then be used to determine PA_*chr*_ at a large scale while reducing the experimental burden of locating expression reporters on the chromosome. We build upon a previous gene expression model in which the promoter activity measured is proportional to the promoter activity per gene copy (pa) and the gene copy number per cell (*g*) and inversely proportional to the cell volume (v) (Klumpp et al., 2009).

**Figure 1:**
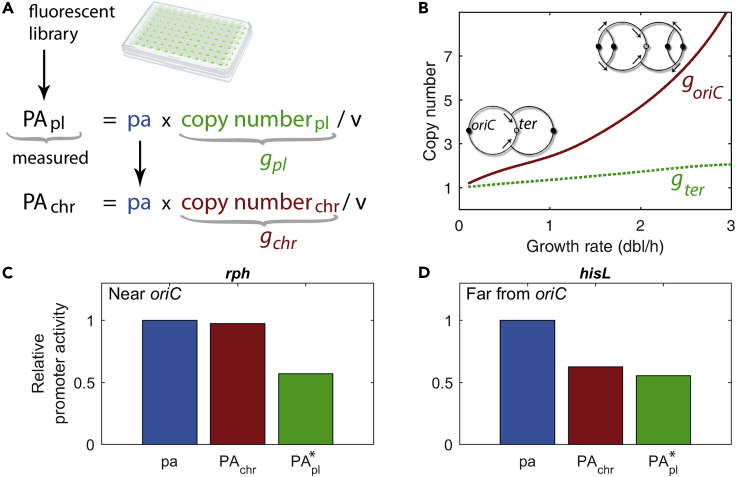
Decoupling Promoter Activity from Gene Copy Number (A) Promoter activity per single gene copy, pa, can be obtained from experimental data of promoter activity quantified with a plasmid library, PA_pl_, once the plasmid copy number *g*_*pl*_ and the growth rate dependence of the cell volume, v, are known. With this, one can calculate the promoter activity of a chromosomal gene, PA_*chr*_, by using Cooper and Helmstetter's model (Transparent Methods). (B) Chromosomal multifork replication makes the copy number per cell of chromosomal genes *g*_*chr*_ dependent on both growth rate and gene location in the chromosome. At a faster growth rate, the number of origins of replication *oriC*s (red solid line and black dots in sketch) increases due to the overlap in time of multiple replication rounds. Arrows show the direction of replication forks. In the case of plasmids with low copy number, as the one used in the plasmid library (pSC101), *g*_*pl*_ is proportional to the number of terminal regions (*ter*s) in the cell (green dotted line). (C and D) Relative differences in promoter activity (pa, PA_*chr*_, PA_*pl*_) for two genes at different chromosomal locations for a fixed growth rate (normalized to the corresponding pa). Genes (*rph* and *hisL*, C and D, respectively) are located at distances *m*_*rph*_ = 0.04 and *m*_*hisL*_ = 0.80 from *oriC*. Observe that chromosomal promoter activity depends strongly on the location of the gene. Data were obtained in balanced growth at μ~0.9 dbl/h (Zaslaver et al., 2009). For comparability, we show PA^∗^_*pl*_ = PA_*pl*_/5 to normalize for the proportionality constant between *g*_*ter*_ and *g*_*pl*_ (see main text for details). See also Figure S1.

We first decouple the copy number signal of the plasmid *g*_*pl*_ that contributes to PA_*pl*_ (Figure 1A). As the replication of these plasmids is synced to the end of the cell cycle (del Solar et al., 1998, Morrison and Chattoraj, 2004), the plasmid copy number *g*_*pl*_ is proportional to that of terminal regions in the chromosome (*g*_*ter*_). Moreover, in the context of this fluorescent library, earlier experimental results showed that proportionality between *g*_*pl*_ and *g*_*ter*_ is equal to 5 independently of both growth rate (up to ∼1.8 dbl/h) and measurement approach (balanced growth and time series) (Gerosa et al., 2013). We can thus consider Cooper and Helmstetter's model (Cooper and Helmstetter, 1968) describing the copy number of a chromosomal gene *g*_*chr*_ for a given growth rate μ ($g_{chr}=2^{\mu \left[C\left(1-m\right)+D\right]}$, *m* represents the normalized distance to the origin of replication of the gene) to obtain the plasmid copy number: $g_{ter}\propto g_{pl}=5\cdot 2^{\mu D}$; the values of *C* and *D* are obtained by interpolation from experimental measurements (Bremer and Dennis, 1996).

Second, with our growth-rate measurements, we decouple the growth-rate-dependent cell size v(μ) with the cell size law that reads $v\left(\mu \right)=2^{\mu \left(C+D\right)}$ in units of unit cell size, and that robustly predicts cell volume under several perturbations (Si et al., 2017). Therefore, from optical density, which is proportional to the total cell mass and volume (Donachie and Robinson, 1987, Nanninga and Woldringh, 1985), we can differentiate whether larger optical density values stem from an increased cell number or cell volume.

This enabled us to compute promoter activity per gene copy, $pa=PA_{pl}v/g_{pl}$, where the effect of gene copy number and volume is excluded, and chromosomal promoter activity $PA_{chr}=PA_{pl}g_{chr}/g_{pl}$_,_ where both effects are included (Figures 1A and 1B). Figures 1C and 1D show the resulting promoter activities of two example cases using experimental data from Zaslaver et al. (2009): genes *rph* and *hisL* located at distances from the origin of replication of $m_{rph}=0.04$ and $m_{hisL}=0.80$, respectively. Differences in chromosomal promoter activity become relevant when comparing genes at different positions in the chromosome. In this way, the distinction between the promoter activity per gene copy (pa, Figure 1A) and chromosomal promoter activity (PA_*chr*_, Figure 1A) emphasizes the added effects of multifork replication depending on the location of the gene. Note that, due to the increase in copy number, the level of PA_chr_ of promoters closest to *oriC* keeps up with the increase in cell volume. We further tested our model by comparing the relative activities of three genes of interest: *maoP*, *pyrB,* and *racR* located at distances *m* = 0.01, *m* = 0.24, and *m* = 0.92, respectively, relative to *oriC*. We find that PA_*chr*_ computed with our model predicts better the relative transcription levels obtained by RT-qPCR than PA_pl_ (Transparent Methods; Figure S1 in the Supplemental Information).

### Constitutive Genes Show a Promoter-Specific Hyperbolic Response to Global Regulation

We applied the previous approach to characterize the global program at a large scale. Constitutive genes appear as the most suitable model given the absence of any specific regulation acting on them, and a list of these genes can be proposed with the information available in current databases (Transparent Methods; Discussion). However, characterizing the response of constitutive promoters in a traditional manner, i.e., from balanced growth measurements in different carbon sources, limits the scalability of the approach. We follow then here an alternative method and consider instead measurements of promoter activity during dynamic changes of growth rate in a specific carbon source. Note that these measures, in the case of constitutively expressed genes, correlate well with those observed under balanced growth in different growth media (Gerosa et al., 2013).

We thus processed the time series data of the set of 708 “constitutive” genes of *E*. *coli* included in the fluorescent library (Zaslaver et al., 2009) (Transparent Methods). Instead of measuring hundreds of genes in many distinct carbon sources, we considered data during exponential and late-exponential growth (within the first 5 h) in glucose medium supplemented with amino acids to obtain profiles of instantaneous promoter activity and growth rate; PA_*pl*_(μ) profiles (Transparent Methods; Figure S2 in Supplemental Information). Data derived in this way can be decoupled from their plasmid context to get chromosomal, PA_*chr*_(μ), and per gene, pa(μ), profiles (previous section).

**Figure 2:**
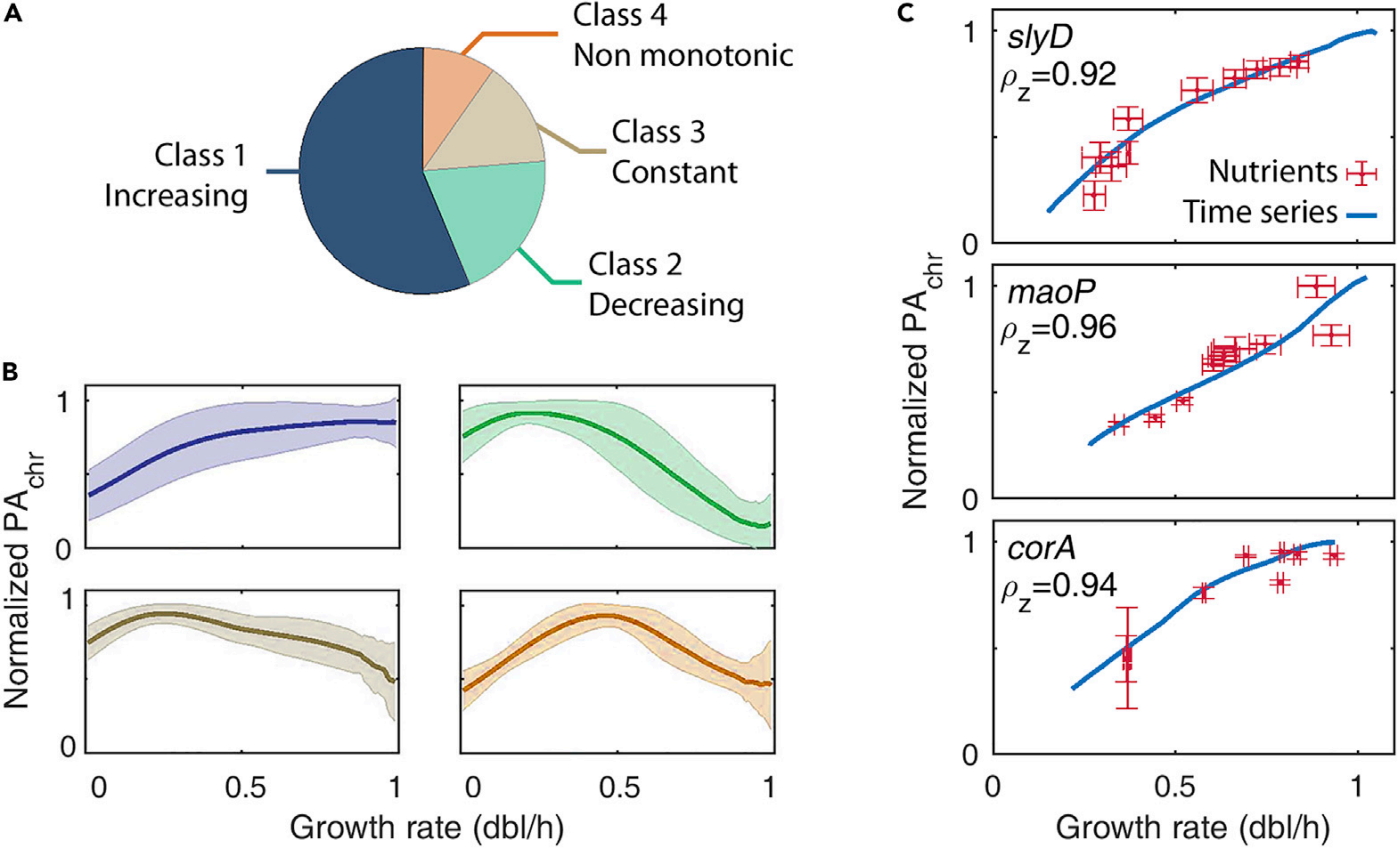
The Clustering Algorithm Groups the PA_chr_(μ) Profiles into Four Classes, of Which Only the First Could Be Validated Experimentally (A) Fraction of promoters found in each of the four classes. Using a clustering algorithm, we grouped the PA_chr_(μ) profiles of about 700 genes with no known TF regulation into four classes following their growth rate dependency. Only class 1 comprises profiles with the expected behavior from earlier works. (B) Mean profile of each class (solid line) and one standard deviation (shaded). Note that these profiles were obtained from time series on a single growth medium (Transparent Methods; Figures S2 and S3). (C) Experimental measurements of PA_chr_ from balanced growth in 10 different media (red crosses, mean and SD from three replicates) validate our approach of inferring the profiles from time series data in glucose supplemented with amino acids (blue solid line, Transparent Methods) of genes in class 1. We also find large linear correlations ρ_z_ between our own time series data and that of Zaslaver et al. (2009). Figure S4 in Supplemental Information shows the experimental results for all 12 promoters tested (three from each class). Data of *corA* grown in glycerol and arabinose resulted in fluorescence levels below the background and are not shown. See also Data S1.

After computing PA_*chr*_(μ) we applied a clustering algorithm that grouped all resulting profiles into four classes (Transparent Methods; cophenetic correlation coefficient c = 0.80, Figures 2A and 2B). Class 1 corresponds to promoters whose activity increases following the expected Michaelis-Menten profile with distinct parameters (Data S1 in Supplemental Information), as it is expected from earlier works (Gerosa et al., 2013, Kochanowski et al., 2017, Liang et al., 1999), whereas classes 2 and 3 correspond to promoter activities that decrease or remain mostly constant across growth rates, respectively. Finally, class 4 includes promoters with a non-monotonic profile that has maximum promoter activity at intermediate growth rates. These classes are robust whether PA_*chr*_(μ) or pa(μ) profiles are used for the classification (Figure S3 and Data S1).

To test the approach of inferring PA_*chr*_ profiles from time series on a single growth medium, we experimentally measured the promoter activity profiles PA_*chr*_(μ) of 12 promoters—chosen among all four classes—from balanced growth data in 10 different growth media (Transparent Methods; Figure S4). The method appeared only particularly robust for all three promoters of class 1, which includes 56% of the total “constitutive” genes considered. Indeed, Figure 2C shows the experimental results of three genes within the first class, namely *slyD*, *maoP,* and *corA* (a brief description of these genes is available in the Transparent Methods section). For genes in classes 2–4, not only do we not recover experimentally the cluster profiles but also we fail to recover the expected Michaelis-Menten hyperbolic pattern of constitutive genes.

In addition, to verify if this lack of signal could be related to the reliability of the clustering algorithm, we added random noise to the chromosomal promoter activity profiles and measured the mean number of recovered genes to the original classification expressed in percentage (10 realizations; Transparent Methods). For normally distributed relative levels of noise up to 10%, we recovered 93% promoters assigned to class 1, whereas the rest of the classes had recovering rates between 35% and 79%. This suggests overall that the discrepancies that we find with classes 2–4 are not related to the approach itself, but rather that these promoters might experience some unknown specific regulatory mechanisms.

However, the robustness with which class 1 promoters are identified and characterized suggests that promoters in this class are likely constitutive. For this reason, we discard promoters from classes 2–4 in the following and use only high-confidence profiles from class 1. Moreover, these results also suggest that as observed previously, during the first hours after balanced growth and before stationary phase, their expression can be well determined by the physiological state of the cell (Gerosa et al., 2013).

### Promoters Sensitive to Global Regulation Are Located Closer to the Origin of Replication

**Figure 3:**
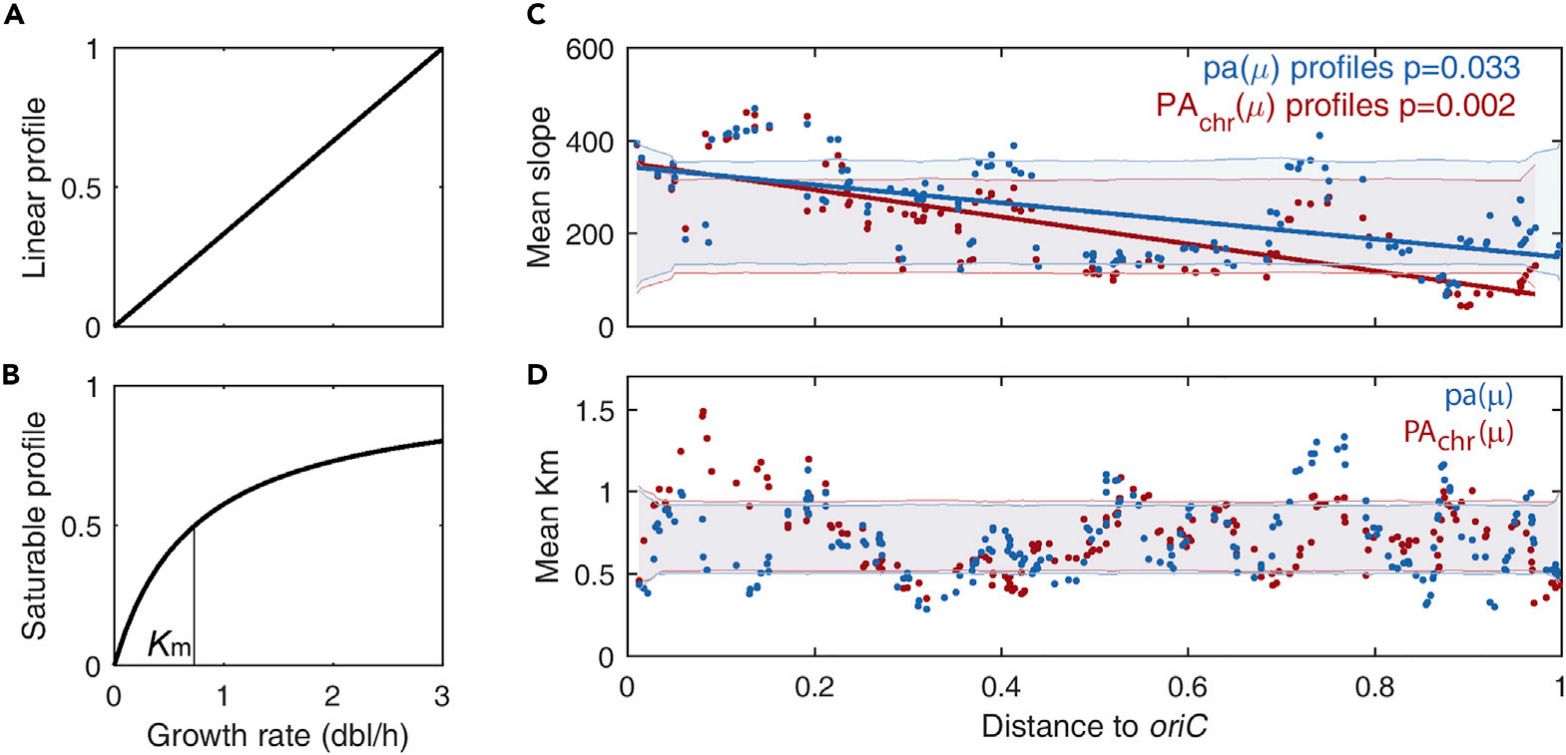
Promoters That Are Most Sensitive to Growth Rate Are Located Closer to the Origin of Replication (A and B) Two profiles of promoter activity can be identified: linear (A) and saturable (B). Sensitivity to the global program is proxied by the slope in the case of linear profiles and the growth rate at which activity is half maximal (*K*_*m*_) in the case of saturable profiles (Figure S5 and Data S1). (C and D) Running averages of the sensitivity to the global program of promoters with linear (C) and saturable (D) profiles, of PA_*chr*_ (red dots) and pa (blue dots). The sensitivity of linear profiles decreases linearly with the distance to *oriC* (solid lines); this pattern is only significantly observed in saturable profiles when considering PA_*chr*_. Shading denotes one standard deviation of sensitivities obtained from a permutation test with 10^4^ randomizations (Transparent Methods).

Beyond the previous classification, we noted different genes within class 1 promoters with distinct sensitivity to the global regulation. To quantify sensitivity, we fitted PA_*chr*_(μ) profiles to a Michaelis-Menten equation:

(Equation 1.1) $$PA_{chr}=\frac{V_{m}\mu }{K_{m}+\mu }\text{,}$$

where *V*_*m*_ is the maximum promoter activity and *K*_*m*_ is the growth rate at which PA_*chr*_(μ) is half maximal; note that μ records the global program and that the different responses emphasize a promoter-specific rather than an unspecific pattern (Gerosa et al., 2013, Klumpp and Hwa, 2008, Liang et al., 1999). Next, we classified profiles with *K*_*m*_ > 3 dbl/h and 0.1 < *K*_*m*_ < 3 dbl/h as linear (Figure 3A) and saturable (Figure 3B), respectively (Transparent Methods). The classification is robust with respect to different thresholds within realistic growth rates; only a small number of genes, around 7% for both pa and PA_*chr*_, have *K*_*m*_ values within 2 and 4 dbl/h. In addition, within this range, the presence and significance of the signals shown in Figure 3 are qualitative and quantitatively robust.

In the case of promoters with linear profiles, we defined the sensitivity to the global program as the slope of the PA_*chr*_(μ) profile, such that larger values stand for larger increases in promoter activity for fixed changes in growth rate. In the case of saturable promoters, we took *K*_*m*_ as a proxy of their sensitivity to the global program: for smaller values of *K*_*m*_ the promoter activity becomes near saturation at smaller growth rates, thus becoming less sensitive to changes in growth rate. We also computed sensitivities of pa(μ) profiles determined in an analogous manner (Transparent Methods; Figure S5 and Data S1).

We then asked if there exists an association between sensitivity and chromosomal location, given that one of the factors that influence these responses is multifork replication, relevant near *oriC*. Figures 3C and 3D show the running average of the sensitivities to the global program along the chromosome of constitutive promoters with linear and saturable profiles, when including and excluding the effects of multifork replication, i.e., PA_*chr*_(μ) and pa(μ), respectively. We observed that the sensitivity of linear profiles decreases linearly with the distance to *oriC* more abruptly and more significantly when considering PA_*chr*_ than pa. In the case of saturable constitutive promoters, we notice that only when considering PA_*chr*_ there is a significant peak within *m* < 0.20 of the chromosome (p < 0.05).

In general, these results suggest that saturable promoters in *E*. *coli* are located across the genome independently of their promoter activity per gene copy. On the contrary, linear promoters that are most growth rate dependent are preferentially located near the origin of replication where they can further boost their expression due to increased copy numbers at large growth rates.

### Global Regulation Acts as a Gene Position Conservation Force

In light of the previous results, it is reasonable to hypothesize that both modes of regulation (gene location, and the sensitivity to the global program) would act synergistically in species experiencing multiple overlapping replication rounds, hence preserving gene order. Inversely, gene order should be lost only in species living in rather stable environments or experiencing long doubling times.

**Figure 4:**
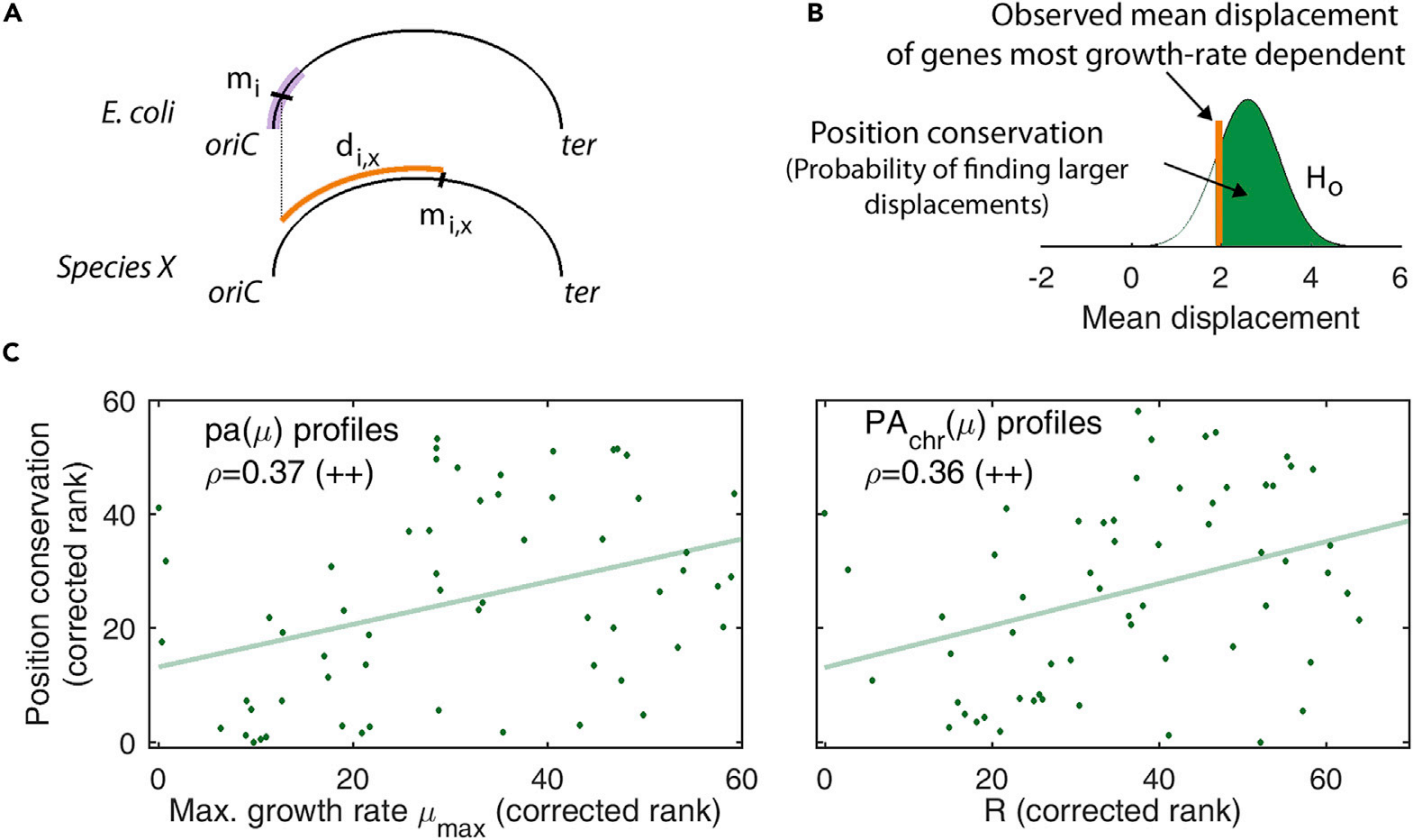
The Position Conservation of Constitutive Genes near *oriC* that Are Most Dependent on the Global Program Correlates with the Maximum Growth Rate and R (A) Gene's position conservation is computed from the displacement of a gene in *E*. *coli* (*m*_*i*_) within the *m* < 0.2 region (purple), with respect to its homolog in other species (*m*_*i*,*X*_). (B) In every species, the observed mean displacement of genes that are most dependent on the global program and are located at *m*=<0.20 is tested against the displacement of the rest of constitutive genes at *m*=<0.20 (H_o_). (C) The most predictive partial correlations (Spearman ρ, and light green solid line, both p < 0.01, denoted as ++) of the position conservation of the half most growth-rate-dependent lineal profiles near *oriC* in *E*. *coli* were obtained with R, for PA_*chr*_(μ) profiles, and the maximum growth rate, for pa(μ) profiles. Variables are corrected for phylogenetic inertia (Transparent Methods; Figures S6–S9 and Data S2).

To evaluate this hypothesis, we examined next if these genes maintain their proximity to *oriC* in other species as a function of some characteristics of the species: their maximum growth rate, the variability of the environment where they live, and the capacity for multifork replication (a function of genome size and minimal doubling time). We performed a homolog search across 100 species to compute the corresponding chromosomal displacement (Figure 4A; Transparent Methods). Displacements of the half most growth-rate-dependent genes near *oriC* (*m* < 0.2; Figure 3) are compared against the null hypothesis, i.e., displacement is independent of sensitivity. This is scored by the probability of finding a larger mean displacement of gene groups of the same size chosen randomly among all constitutive promoters at *m* < 0.2, for linear or saturated growth rate dependencies (Figure 4B). Smaller values of this score, termed the position conservation, represent non-conserved locations of promoters.

We studied next the association between the position conservation and three main species features: environmental variability (env), relevance of multifork replication (R), and maximal growth rate (as the inverse of the minimal doubling time, μ_max_ = τ_min_^−1^). Environmental variability was based on an earlier environmental classification (Parter et al., 2007), whereas minimal doubling time with genome size was estimated to compute R, the ratio between the maximal chromosome's replication time and the minimal doubling time as a measure of the importance of multifork replication effects for an organism (Couturier and Rocha, 2006). For each class of promoter dependence (linear and saturated, pa and PA_*chr*_) we measured the partial Spearman's rank correlation ρ between the corresponding position conservation and env, R, or μ_max_ while controlling in all cases for phylogenetic distance (Transparent Methods).

In the case of lineal promoters, we obtained a significant correlation between position conservation and R or μ_max_ (all Spearman ρ with p < 0.01). The numerical values of ρ are in line with those obtained in other gene order studies (Couturier and Rocha, 2006). Correlations with R are only slightly stronger when the global program includes the multifork effect (PA_*chr*_; 0.36 vs. 0.35), as expected from the definition of R, whereas maximal growth rate and R are equally relevant when not including the multifork dosage effect (pa; 0.37 versus 0.37). Figure 4C explicitly shows these correlations: pa(μ) versus maximum growth rate and PA_*chr*_(μ) versus R. The position conservation of saturable promoters was not significant in any case.

Overall these results support our hypothesis that the impact of the global transcriptional program on gene order is a general feature of bacterial species, especially in those that undergo multifork replication. In fact, the position of genes exhibiting a particularly sensitive linear response tends to be conserved in species with larger values of R and fast growth rates.

## Discussion

We quantified growth rate dependencies of over 700 prospective constitutive genes in *E*. *coli*, arguably the best gene collection to explore the effects of the physiological state of the cell, the global program, on gene expression. This is based on an approach that obtains promoter activity as if reporters had been inserted in the chromosome and that characterizes growth rate dependencies from dynamical data. Both features reduce the costs and difficulties of large-scale experiments.

Half of the promoters that we examine present a Michaelis-Menten rate law confirming earlier reports (Gerosa et al., 2013, Liang et al., 1999). That we verify this class with experiments in which the dependency is obtained using conventional approaches (growth rate being modified with the utilization of different carbon sources; data obtained at steady state) supports our approach. However, we also find three other patterns that differ. This does not seem to be associated with the method itself as our experimental characterization of these responses did not recover hyperbolic profiles. These genes could be perhaps subject to additional layers of regulation or other hidden structural aspects, which in turn makes us expect the lack of correlations between balanced and dynamic growth measurements (Figure S4). However, we observed no signal of a particular enrichment on specific sigma factors or AT content in the promoter region or upstream of it (Figures S6A and S6B in Supplemental Information), as large AT content is known to favor DNA bending and thus protein-DNA interactions (Dorman and Dorman, 2016, Mitchison, 2005), in particular, upstream of the promoter region in the UP element (Estrem et al., 1998). In addition, although the supercoiling state of the chromosome is known to affect gene expression, no quantitative or even qualitative genome-wide regulatory model is yet available (Lal et al., 2016). We considered instead data on independent supercoiling macrodomains (Valens et al., 2004) to notice again no signal (Figure S6C in Supplemental Information).

Overall, the success in predicting the response of over 50% promoters with the original list (arguably the truly constitutive ones) demonstrates the significance of the global program beyond balanced growth (Berthoumieux et al., 2013). Within these promoters we distinguish subsets that are especially sensitive to growth rate and that are selectively located in the chromosome. Indeed, genes with either linear or saturable profiles show larger sensitivities to growth rate within 20% of the replichore closest to *oriC*. This pattern is partially maintained when we control for the multiple replication fork effect, i.e., when we consider pa(μ) profiles instead of PA_*chr*_(μ). We thus propose a model in which multifork effects and the global program (excluding gene copy) work in combination: promoters that are most growth rate dependent in *E*. *coli* benefit from a larger increase in gene expression at large growth rates (Figure S7 in Supplemental Information).

However, the fact that *E*. *coli* coordinates different mechanisms to obtain a multiplicative effect of enhanced expression of genes near *oriC* might not necessarily be a general property of bacterial genomes. This precise coupling might have been selected in bacteria for which multifork gene dosage fluctuations are relevant: those that are subject to variable growth rates or bacteria that reach a large number of overlapping replication rounds. We found evidence that supports our hypothesis: gene order conservation of the most sensitive genes to the global program correlates significantly with the potential relevance of multifork replication in over 100 species. In addition, a recent study found two fundamental bacterial reproduction strategies, the first relying on (metabolically) efficient but slow growth and a second that relies on inefficient but fast growth (Roller et al., 2016). Of the two strategies, the latter perhaps exploits the coordination of these mechanisms. Also, correlations involving maximal growth rate should be taken cautiously as known doubling times are biased by laboratory-controlled environments (Gibson et al., 2018).

Recent studies show the important link between gene expression and gene location on the chromosome (Block et al., 2012, Bryant et al., 2014). Indeed, the increase in gene dosage due to bacterial multifork replication appears as an added control mechanism of natural genetic circuits (Bar-Ziv et al., 2016, Slager and Veening, 2016). However, the relevance of genome organization goes beyond gene dosage fluctuations in fast growth (Sobetzko et al., 2012, Soler-Bistué et al., 2017), and it may be influenced by chromosomal structure (Sobetzko et al., 2012) and gene essentiality (Rocha and Danchin, 2003).

Our work builds on these studies to emphasize the genome-wide effect of the physiological state of the cell (the global program) on the control of gene expression and its coupling to genome organization. In fact, not only do we find that promoters that are most growth rate dependent (at a single copy level) are located significantly close to *oriC* in *E*. *coli* but also that this feature is conserved in species for which multifork gene dosage fluctuations are strongest. Therefore, we present the physiological control of gene expression as an additional aspect to consider if we are to elucidate the organization and evolutionary dynamics of the bacterial genome.

### Limitations of the Study

In this study we showed that promoters whose transcriptional response is more dependent on growth rate are preferentially located closer to the origin of replication in the chromosome in *E*. *coli*, and that the relative location of these genes in other species correlates significantly with their respective growth dynamics, directly related to their habitat. One limitation of the study is that it relies on mean, population-level data of transcriptional expression, as the experiments are performed in batch culture. Data on single-cell transcriptional expression variability could further be of interest but are limited by the scalability of the experimental setup. Finally, the homologs of *E*. *coli*'s genes in other species might have differences at the promoter level: different affinity to the RNA polymerase or the acquisition of regulatory sequences of TFs. This is again beyond reach due to the scale of the experiments required.

## Methods

All methods can be found in the accompanying Transparent Methods supplemental file.

## Data and Code Availability

Data and code are available upon request to the authors.

## Supplemental Information

Document S1. Transparent Methods and Figures S1–S9

Data S1. List of Genes with Their Corresponding Classes and Best-Fit Parameters, Related to Figures 2 and 3

Data S2. List of Species with Their Corresponding Parameters, Related to Figure 4

## Footnotes

Supplemental Information can be found online at https://doi.org/10.1016/j.isci.2020.101029.
