# Supplementary material for: The Impact of Global Transcriptional Regulation on Bacterial Gene Order
Source: iScience. 2020 Apr 2;23(4):101029. doi: 10.1016/j.isci.2020.101029 (PMC7155222; doi:10.1016/j.isci.2020.101029)
Supplement: Document S1. Transparent Methods and Figures S1–S9 [file mmc1.pdf]

iScience, Volume *23*

Supplemental Information

The Impact of Global Transcriptional Regulation on Bacterial Gene Order

Pablo Yubero and Juan F. Poyatos

Supplemental figures and legends

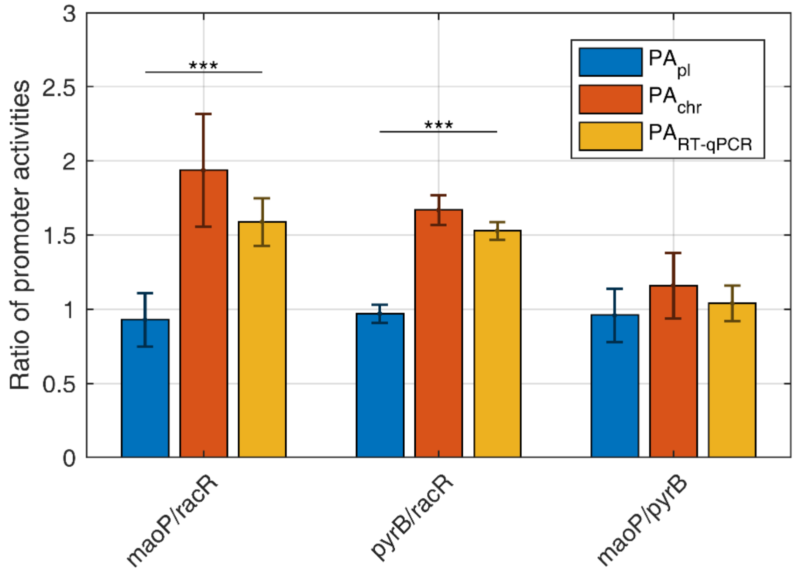

Fig. S1. Chromosomal promoter activities are anticipated by our model from measurements of a fluorescent reporter plasmid library, related to Figure 1. We find that the relative promoter activities obtained from RT-qPCR are better anticipated by our model than from fluorescent plasmid reporters (asterisks denote rejection of the null hypothesis of equal means of a two-tailed, two sample t-test with a confidence level of α=0.001). Additionally, and as expected from the model, RT-qPCR data of PA ratios is well fitted by an exponential decreasing function of chromosomal distance between genes (non-linear least squares, R^2=0.90) unlike data from fluorescent reporters (R^2=-0.06). Fits not shown. RT-qPCR values are the mean of three biological replicates, and error bars denote two standard deviations (Methods).

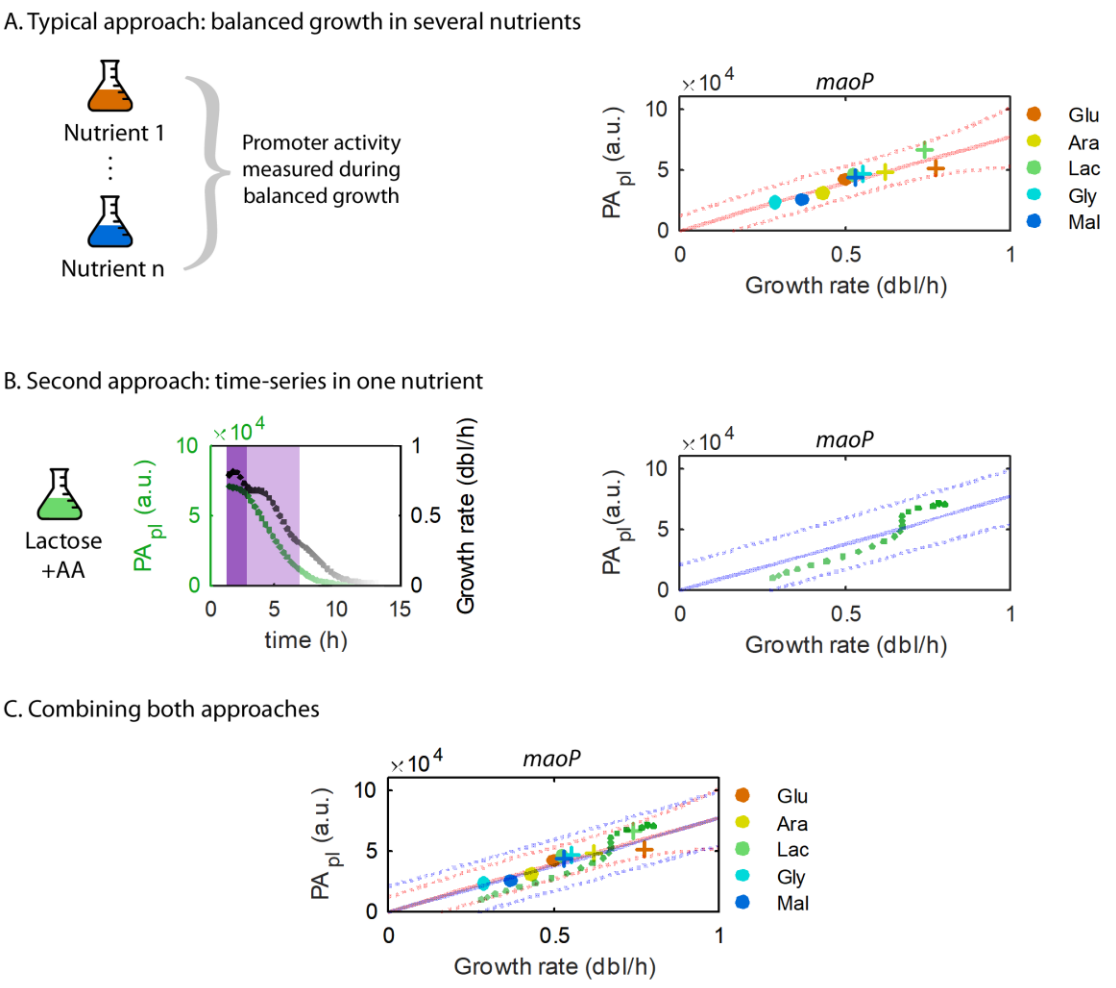

Fig. S2. **Comparison between two different approaches to compute promoter activity profiles, related to Figure 2.** (**A**) The traditional approach consists in obtaining data pairs (PA_{pl}, μ) from cultures during balanced growth in a variety of growth media. Experimental profile obtained for the promoter of gene *maoP* in minimal medium with five different carbon sources: glucose (orange), arabinose (yellow), lactose (green), glycerol (light blue), maltose (dark blue) and supplemented, or not, with amino acids (AA; crosses and circles respectively; Materials and methods). The best fit to Eq.(1) is also shown with the 95% confidence interval (red solid and dotted lines respectively). (**B**) We can also obtain promoter activity profiles from growth time-series in a single growth medium (e.g. lactose+AA). We consider promoter activity (in green) and growth rate (in black) points during early and late exponential phase (dark and light purple shade) to obtain a similar promoter activity profile (Materials and methods). The best fit to Eq. (1) and the 95% confidence bounds are also shown (blue solid and dotted lines respectively). (**C**) Superposition of the two profiles obtained previously by different means. These two approaches yield similar qualitative and quantitative profiles in this and other cases for promoters of genes in class 1.

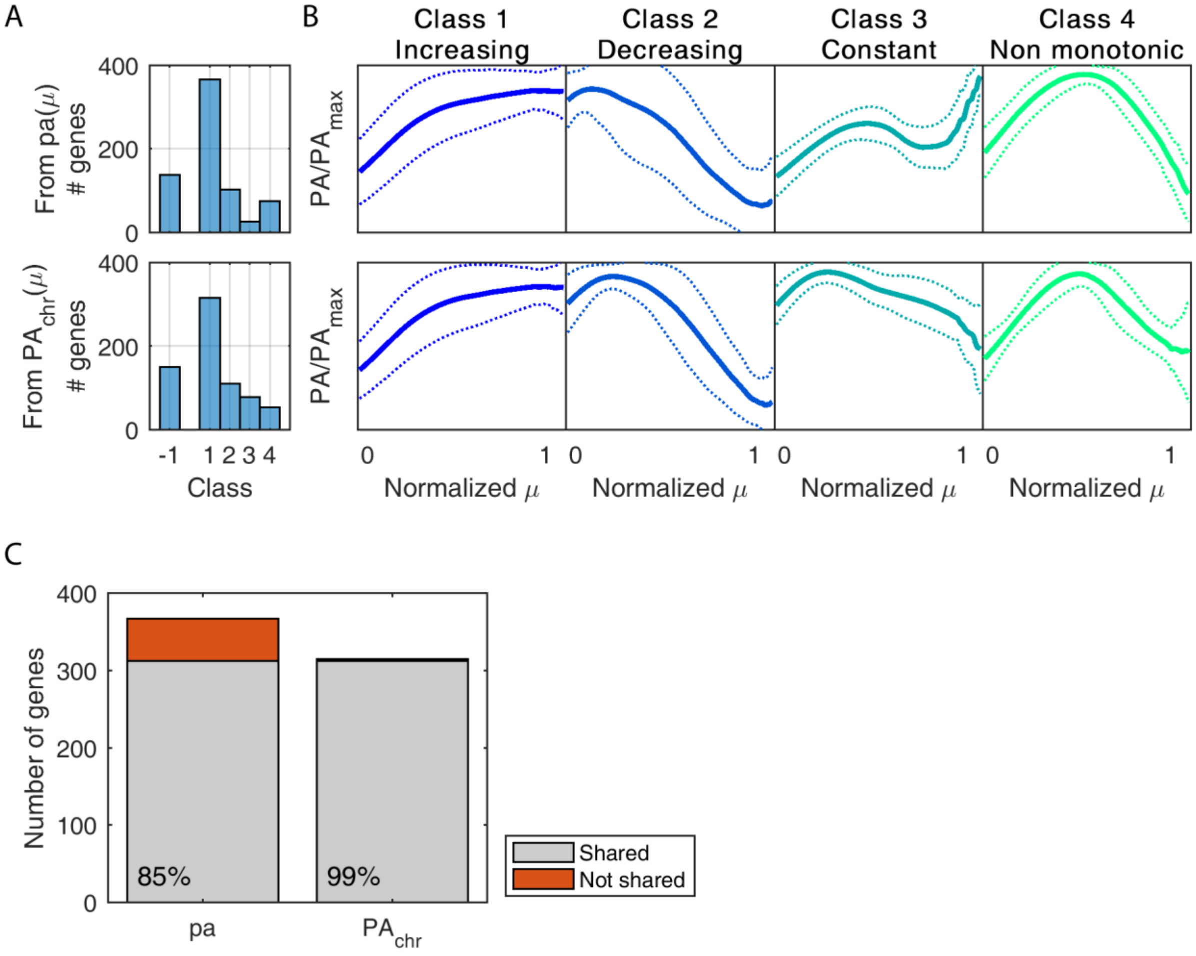

Fig. S3. Comparison of the results of the automatic clustering algorithm when considering pa(μ) and PA$_{chr}$(μ) profiles, related to Figure 2. (**A**) Histograms with the number of promoters found in each class. Class -1 includes all discarded promoters during the clustering algorithm (Materials and methods). (**B**) Mean profiles (solid lines) with one standard deviation (dotted lines) of each class. (**C**) The composition of class 1 is robust whether computed from pa(μ) or PA$_{chr}$(μ) profiles. In fact, 85% (312 out of 367) and 99% (312 out of 315) of genes are shared, respectively.

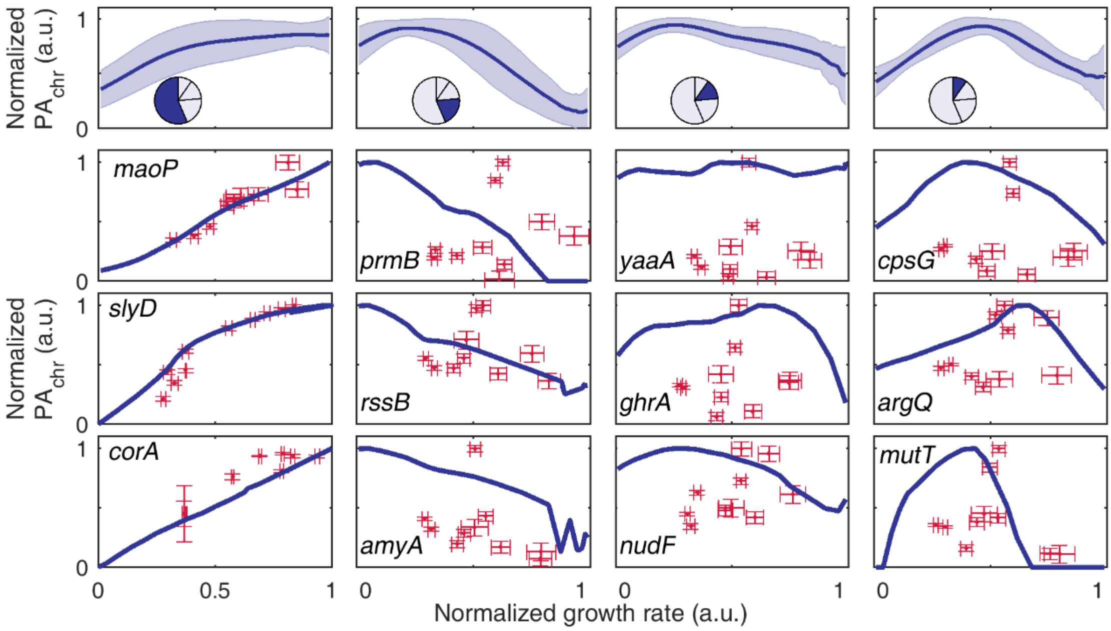

Fig. S4. The experimental profiles obtained from balanced growth in ten different media and time series in a single growing medium correlate only for genes in class 1, related to Figure 2. (**A**) Mean $PA_{chr}(\mu)$ profile (blue line, shaded area represents one standard deviation) of each of the four classes obtained from the clustering algorithm (Materials and Methods). Inset: pie chart with the fraction of promoters found in that particular class. (**B**) Experimental measurements of PA_{chr} from balanced growth in ten different media (red crosses, mean and sd from three replicates) of 12 genes (three from each class). Profiles from time series data in glucose supplemented with amino acids are superimposed in blue solid line [Zaslaver *et al*. (2009)]. Observe that the expected hyperbolic pattern for constitutive genes is only recovered for those in class 1. Data of *corA* grown in glycerol and arabinose resulted in fluorescence levels below the background and are not shown.

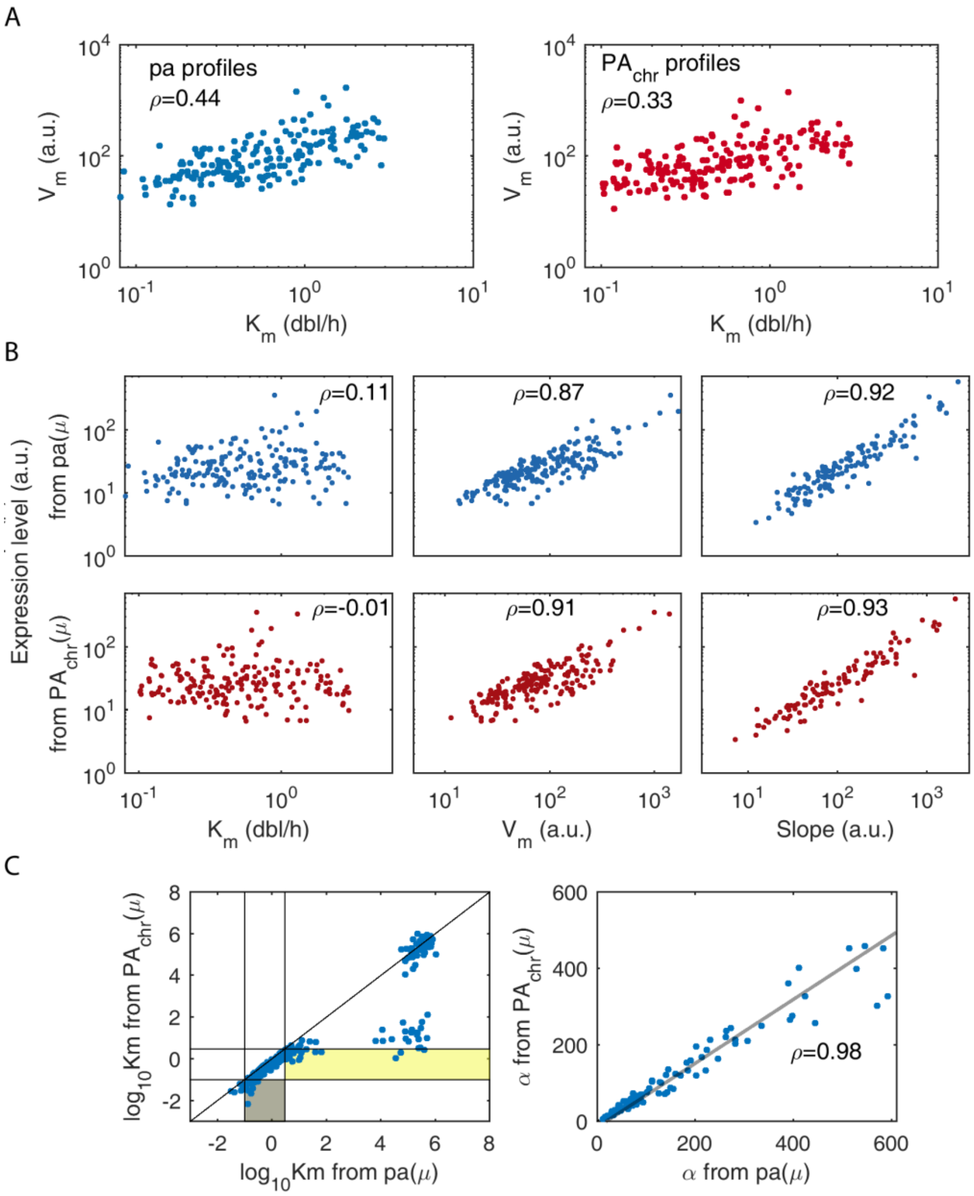

Fig. S5. Correlation between the parameters obtained from fitting pa(μ) and PA$_{chr}$(μ) to Eq.(1), related to Figure 3. (**A**) There is a small correlation between the strength of a saturable promoter (V_m) and its sensitivity to growth rate (K_m). (**B**) The expression level of a promoter, measured as its activity at μ=0.5 dbl/h, does not correlate with the sensitivity of saturable promoters K_m. Predictably, expression level correlates well with the maximum activity of saturable promoters, and with the slope of lineal promoters. (**C**) Different sensitivities are obtained from pa(μ) and PA$_{chr}$(μ) profiles. Left: values of K_m of the 312 promoters belonging to class 1 when computed from pa(μ) and PA$_{chr}$(μ). The region where promoters whose pa is saturable (non-saturable) but whose PA$_{chr}$(μ) is constant (saturable) is marked in grey (yellow). Right: slopes of linear profiles computed from pa and PA$_{chr}$ correlate linearly. In all panels, ρ is Pearson's linear correlation coefficient, and a log-log scale is used for clarity because the values span several orders of magnitude.

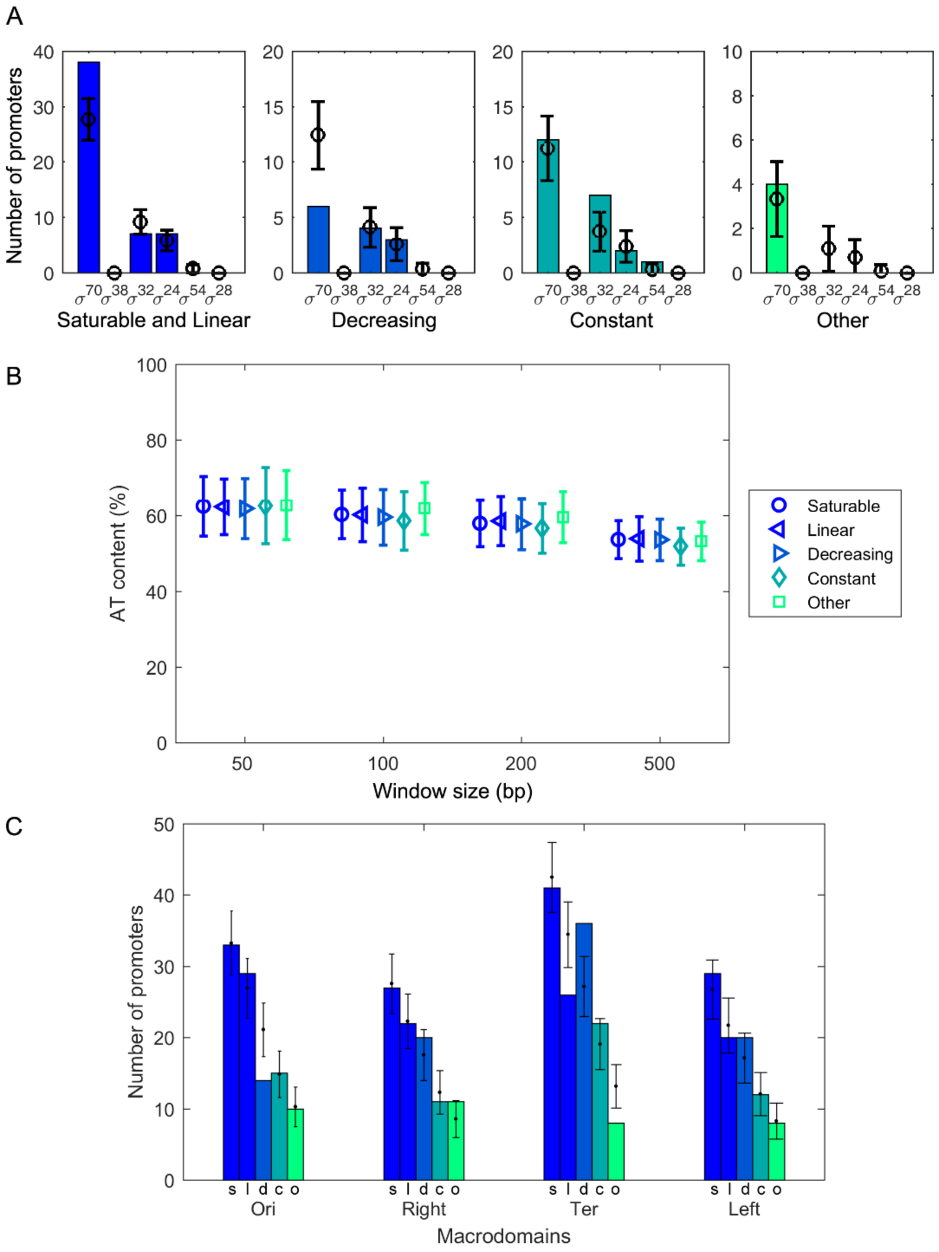

Fig. S6. Sigma factors, AT content and macrodomains do not explain the different classes, related to Figure 4. (**A**) Number of observed genes (vertical bars) within each sigmulon (x-axis) and the expected values under 10^4 randomizations (black circles, error bars correspond to one standard deviation; housekeeping -σ70-, general stress - σ38-, cytoplasmic stress - σ32-, extracytoplasmic stress - σ24-, nitrogen stress - σ54- and flagellar genes - σ28-). (**B**) Mean AT content and one standard deviation (y-axis) in different windows of the upstream region of the initiation transcription site (x-axis). Although constitutive promoters are often considered to be about 100bp long, we show the results of different window sizes. (**C**) Number of promoters of each class found in the chromosomal macrodomains (colored bars; classes: saturable -s-, linear -l-, decreasing -d-, constant -c-, and other -o-). Black points and error bars correspond to the mean and one standard deviation of the expected number of promoters found under 10^5 randomizations of their location.

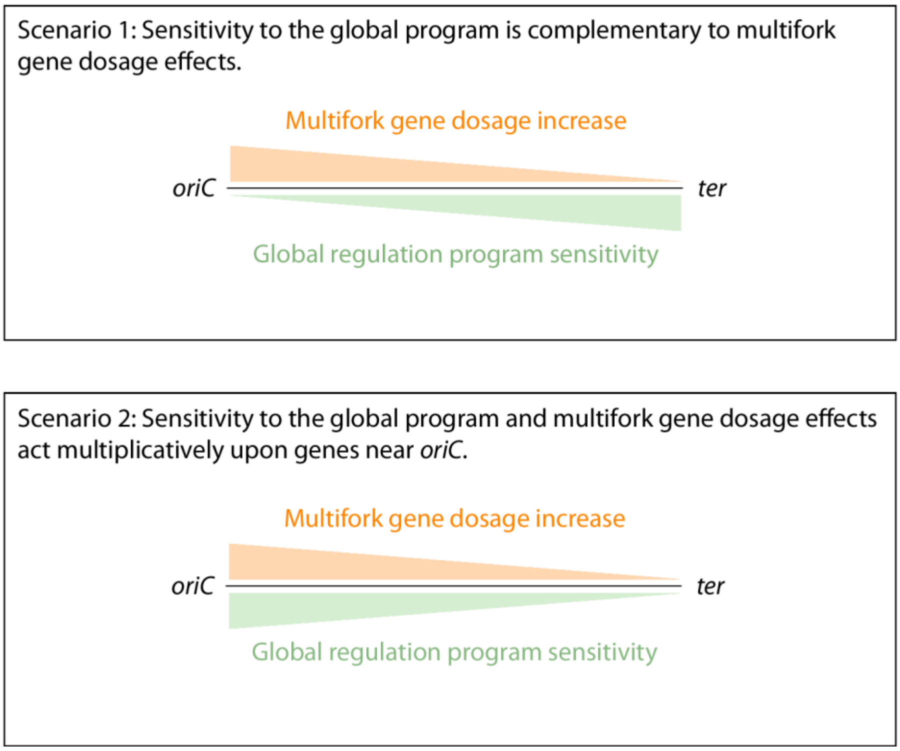

Fig. S7. Two possible scenarios for the effect of multifork gene dosage as part of the global program, related to Figure 4. (**Top**) Scenario 1. The most sensitive genes to the global program locate near the *ter* region (symbolized by a green gradient; note that global regulation here excluded the impact of gene dosage). In contrast, the multifork effect is stronger near *oriC* (orange gradient). The gene dosage effect is consequently not coupled to a strong sensitivity. (**Bottom**) Scenario 2. The most sensitive genes to the global regulation (excluding gene dosage) locate in the *oriC* region. In this case, the effect of gene dosage is linked to the strong sensitivity.

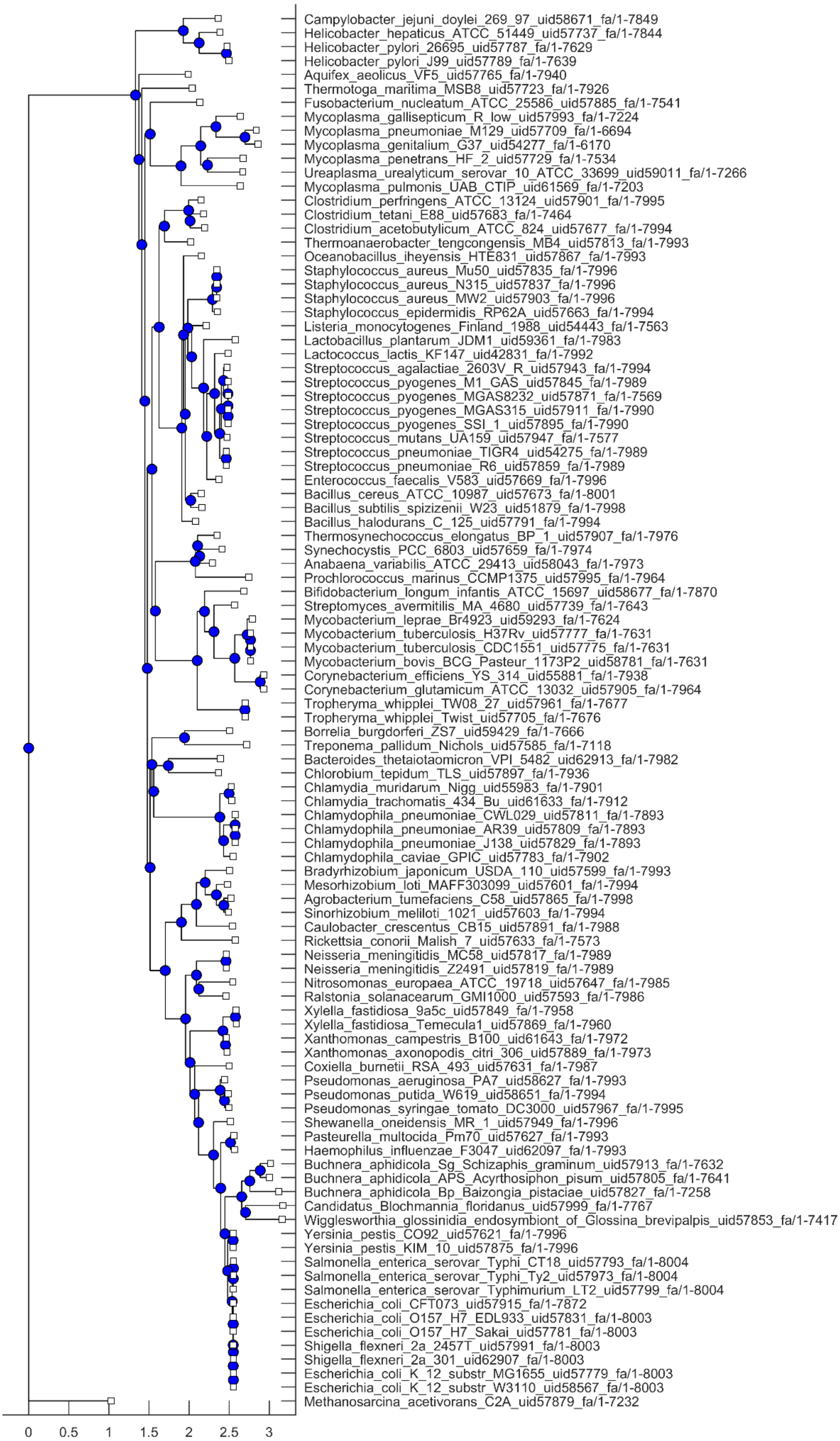

Fig. S8. Phylogenetic tree of the 100 species used in this work, related to Figure 4.

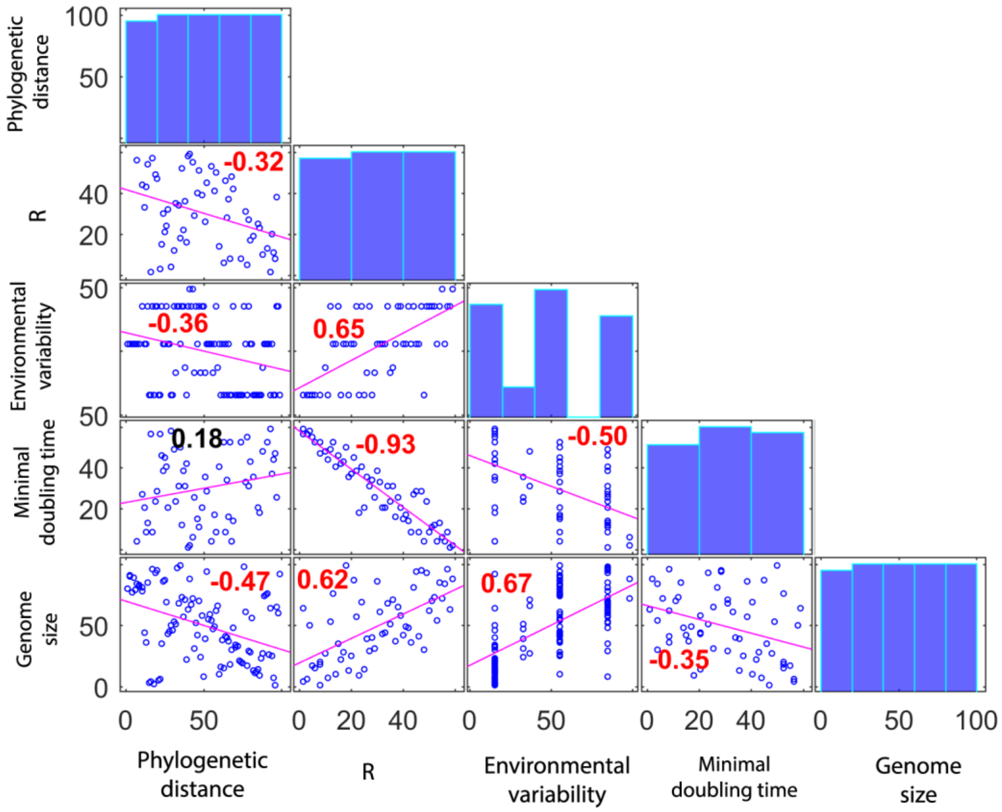

Fig. S9. Spearman rank correlations between possible explanatory variables for conservation measures, related to Figure 4. Correlations (with ties) for all species between phylogenetic distance, R (i.e. the relevance of multifork effects), environmental variability, minimal doubling time and genome size in Mbp, when available (Materials and methods). Values in red denote significant correlations ($p<0.05$). Histograms of the ranks are shown in the diagonal.

Transparent Methods

Promoter activity data and validating experiments

We obtained time series of optical density and promoter activity from public available data (Zaslaver et al., 2009) that used a library of *E. coli* promoters expressing a fast-folding fluorescent protein and cloned in a low-copy plasmid (Zaslaver et al., 2006). We considered only data of those experiments using minimal medium with 0.5% (w/v) glucose and supplemented amino acids and did not include experiments in which the strains did not grow (within one standard deviation of the mean growth curve) or whose promoter activity was constant and equal to zero.

We used this very library (*E. coli* K-12 MG1655 strain) in our validation experiments to test 3 representative promoters from each of the four response classes. The list of their names and their products' are: *maoP* (macrodomain Ori protein), *slyD* (peptidyl prolyl *cis/trans*-isomerase and chaperone), *corA* ($Ni^{2+}/Co^{2+}/Mg^{2+}$ transporter), *prmB* (50S ribosomal subunit protein L3 N5-glutamine methyltransferase), *rssB* (regulator of sigmaS), *amyA* (α-amylase), *yaaA* (peroxide stress resistance protein), *ghrA* (glyoxylate/hydroxypyruvate reductase A), *nudF* (ADP-sugar pyrophosphatase), *cpsG* (phosphomannomutase), *argQ* (tRNA) and *mutT* (8-oxo-dGTP diphosphatase). The reporter strains of these genes were retrieved from frozen stocks, plated in selective media, and grown overnight. Isolated colonies were grown overnight in the specific medium, then diluted 1/20 and pre-cultured for about 5h. Then, 96-well flat transparent plates containing 190µl of the specific medium were inoculated 1/20 with the pre-culture and added 50µl of mineral oil to prevent evaporation. Optical density (600 nm) and fluorescence (535 nm) were assayed in a Victor x2 (Perkin Elmer) at 10min intervals for ~8h (growth at 30ºC with shaking).

Cultures were grown in M9 minimal medium with kanamycin (50µg/ml) to which either glucose, arabinose, lactose, glycerol or maltose was added to a final concentration of 0.5% (w/v). All five carbon media were also supplemented in the second set of experiments with amino acids to a final concentration of 0.2% (w/v), thus making 10 different nutrient conditions in total.

RT-qPCR protocol. We harvested cells growing exponentially in M9+Gluc 0.5%+AA 0.2% at 37ºC by centrifugation at 4000rpm at 4ºC for 10mins to quickly freeze them at -80ºC. The day after, RNA was extracted with Trizol (Invitrogen) and the resulting aqueous phase was directly purified with the RNeasy Mini Kit (Qiagen) and treated with Turbo DNA-free Kit (Invitrogen) following standard protocols. The samples where then handled by the Genomics Service of the CNB to obtain RT-qPCR

results. Forward and reverse primers were: CAACTGCTTG AGCGTCATGG and CCACGGCATA CTGCTACGAA for *maoP*, ATCGCATCGA CGTAAGTGCT and CTTCTCCGAC AGCGCCAATA for *pyrB* and TTAAAGTGGC GTGCGACCTC and AGACTTAGGC CGAGCGATAGA for *racR*. We computed PA_{pl} following the previous protocol for reporter strains but grown at 37ºC instead. Growth rate was similar and about 1.5 dbl/h for all strains (fluorescence assay and RNA extraction).

Data processing and modeling

Growth rate time series were computed as the two-point finite differences of log(OD), $\mu(t)=\Delta\log(OD)/\Delta t$, and promoter activities were computed as the two-point finite difference in time of fluorescence per OD unit, $PA_{pl}(t)=\Delta GFP/\Delta t/OD$. Balanced-growth data was computed from the mean time-series measurements of three technical replicates as the average value in a ~2h time-window during observable exponential growth. Promoter activity is in units of GFP/OD/h. Note that the extensive generality and robustness of the cell size formula derived by Si and colleagues (Si et al., 2017), expressed as $v = 2^{\frac{\tau_{cyc}}{\tau}}$ with $\tau_{cyc} = C + D$ and $\mu = 1/\tau$ by definition in their supplementary material, suggests that it is a good first approximation of the cell size during the early and late exponential phases of growing *E. coli* in standard conditions.

Automatic clustering was performed with the normalized growth rates and promoter activities (PA_{chr} and pa) with respect to their maximum. The euclidean pairwise distance was used to compute the linkage matrix with the unweighted average distance, which was then automatically divided into 50 clusters of which were rejected those with less than 2% of the sampled promoters. Clusters were then grouped together by visual interpretation resulting in the four classes presented in the main text. The robustness of the classification was tested against additional relative random noise of 5% and 10% normally distributed. The recovering rate is the mean number of genes classified as in the original classes expressed in percentage from 10 realizations. Promoter activity profiles of cluster 1 (either from pa and PA_{chr}) were then fit to Eq.1 by means of non-linear least squares method. We fine-tuned the automatic classification and depending on the value estimated, by inter- or extrapolation, for K_m we distinguish between linear (K_m>3 dbl/h), saturable (0.1< K_m <3 dbl/h) and constant (K_m<0.1 dbl/h) profiles. The slope of linear profiles was obtained from linear least squares fits. Figure 3C-D shows the running averages of the sensitivities in a window of 10 genes, and although some oscillatory patterns can be appreciated, that is beyond the scope of this study.

Definition of constitutive genes

We selected constitutive promoters as those lacking any interaction with DNA-binding transcriptional factors, even with weakly specific factors as IHF and H-NS. For this we used the regulatory network of *E. coli* downloaded from RegulonDB (Gama-Castro et al., 2016), arguably the best characterized regulatory network to date of any living organism. This gene list overlaps considerably with the set of the constitutive promoters independently identified by Genomic SELEX screening (Shimada et al., 2017). Moreover, note that the promoter-specific values V_m and K_m can be modulated by factors that bind to RNA polymerase like (p)ppGpp. That the alarmone (p)ppGpp affects the expression of >30% of the genome of *E. coli* (Traxler et al., 2008) denotes its relevance in gene expression control. However, (p)ppGpp's role is beyond specific regulation. In fact, recent results show that it is a leading component for the proper, coordinated regulation of bacterial physiological state (Dennis et al., 2004; Traxler et al., 2008), which is precisely the global program. For this reason, the expression control effects of (p)ppGpp with or without *dksA* are not considered to be part of a specific regulation, as neither does RegulonDB. In addition, that we find 3 ribosomal genes (*rpsT, rpsB* and *rpmE*) in our list highlights the fact that we focus only on transcriptional and not post-transcriptional regulation, because of the limitation imposed by the use of fluorescent reporter measurements.

Environmental variability, R and phylogenetic distances

The classification of over 100 species depending on the variability of their environment used in this work was previously published (Parter et al., 2007). Classes of increasing environmental variability are obligate, specialized, aquatic, facultative, multiple and terrestrial. *E. coli* is found in the facultative class. From the original list, species with more than one chromosome, species that could not be found in the phylogenetic tree (see below), and species without a published unified genome in NCBI database were not considered in this study (Data set S2 in supplemental material). The importance of multifork dosage increase due to multifork replication in a given species, termed R, is obtained as the ratio of chromosomal replication time by the minimal doubling time for each bacterium. In fact, it is proportional to the maximum number of overlapping replication rounds. Values of R and the minimal doubling time were retrieved for 60 of the 100 species (Couturier and Rocha, 2006). Phylogenetic distances from *E. coli* were computed from the phylogenetic tree of (Lang et al., 2013) (Fig. S8 in supplemental material). These variables correlate with each other and specially with phylogenetic distance to *E. coli* (Fig. S9 in supplemental material). For this reason, we used partial linear correlations (Couturier and Rocha, 2006), which considers the correlation between two variables (position conservation and μ_{max} or R) controlling for a third confounding variable: phylogenetic

distance in this case. The corrected values of position conservation, env, μ_{max} and R are the residuals of their respective rank correlations with phylogenetic distance. Hence, correlation between corrected values are not affected by phylogenetic inertia (Fig. 4C).

Origins of replication and homology search

For the location of the origins of replication of most genomes, we used DoriC v7.0 (Gao et al., 2013), a database of bacterial and archaeal genomes available at http://tubic.tju.edu.cn/doric/ -- the update of September 15th, 2017). For genomes for which the origin of replication was not directly available, *Blochmannia Floridanus* and *Methanosarcina Acetivorans*, we used the web-tool that DoriC offers for its identification. The best results had expected values E=0 and E=3e-9, respectively (Data set S2 in supplemental material). Moreover, the replication terminus *ter* was set half the genome length away from the origin of replication as is done in related works. We obtained the homolog sequences (and their location) of the 708 constitutive genes of *E. coli* from Blastp, results with expected values above E=1e-3 were discarded (Pearson, 2013). We quantified the position conservation of the half most sensitive genes to growth rate, located at $m<0.2$, for a given species as the probability of finding a smaller mean displacement in 10^4 random selections among all homologs found at $m<0.2$ (independently of their sensitivity to growth rate). This protocol controls for a possible general conservation of genes near *oriC*, and for different numbers of homologs found in the set of species.

Supplemental References

Couturier, E., Rocha, E.P.C., 2006. Replication-associated gene dosage effects shape the genomes of fast-growing bacteria but only for transcription and translation genes. Mol. Microbiol. 59, 1506–1518. https://doi.org/10.1111/j.1365-2958.2006.05046.x

Dennis, P.P., Ehrenberg, M., Bremer, H., 2004. Control of rRNA Synthesis in Escherichia coli: a Systems Biology Approach. Microbiol. Mol. Biol. Rev. 68, 639–668. https://doi.org/10.1128/MMBR.68.4.639-668.2004

Gama-Castro, S., Salgado, H., Santos-Zavaleta, A., Ledezma-Tejeida, D., Muñiz-Rascado, L., García-Sotelo, J.S., Alquicira-Hernández, K., Martínez-Flores, I., Pannier, L., Castro-Mondragón, J.A., Medina-Rivera, A., Solano-Lira, H., Bonavides-Martínez, C., Pérez-Rueda, E., Alquicira-Hernández, S., Porrón-Sotelo, L., López-Fuentes, A., Hernández-Koutoucheva, A., Del Moral-Chávez, V., Rinaldi, F., Collado-Vides, J., 2016. RegulonDB version 9.0: high-level integration of gene regulation, coexpression, motif clustering and beyond. Nucleic Acids Res. 44, D133-143. https://doi.org/10.1093/nar/gkv1156

Gao, F., Luo, H., Zhang, C.-T., 2013. DoriC 5.0: an updated database of oriC regions in both bacterial and archaeal genomes. Nucleic Acids Res. 41, D90–D93. https://doi.org/10.1093/nar/gks990

Lang, J.M., Darling, A.E., Eisen, J.A., 2013. Phylogeny of Bacterial and Archaeal Genomes Using Conserved Genes: Supertrees and Supermatrices. PLOS ONE 8, e62510. https://doi.org/10.1371/journal.pone.0062510

Parter, M., Kashtan, N., Alon, U., 2007. Environmental variability and modularity of bacterial metabolic networks. BMC Evol. Biol. 7, 169. https://doi.org/10.1186/1471-2148-7-169

Pearson, W.R., 2013. An Introduction to Sequence Similarity (“Homology”) Searching. Curr. Protoc. Bioinforma. Ed. Board Andreas Baxevanis Al 0 3. https://doi.org/10.1002/0471250953.bi0301s42

Shimada, T., Tanaka, K., Ishihama, A., 2017. The whole set of the constitutive promoters recognized by four minor sigma subunits of Escherichia coli RNA polymerase. PLOS ONE 12, e0179181. https://doi.org/10.1371/journal.pone.0179181

Si, F., Li, D., Cox, S.E., Sauls, J.T., Azizi, O., Sou, C., Schwartz, A.B., Erickstad, M.J., Jun, Y., Li, X., Jun, S., 2017. Invariance of Initiation Mass and Predictability of Cell Size in Escherichia coli. Curr. Biol. 27, 1278–1287. https://doi.org/10.1016/j.cub.2017.03.022

Traxler, M.F., Summers, S.M., Nguyen, H.-T., Zacharia, V.M., Hightower, G.A., Smith, J.T., Conway, T., 2008. The global, ppGpp-mediated stringent response to amino acid starvation in Escherichia coli. Mol. Microbiol. 68, 1128–1148. https://doi.org/10.1111/j.1365-2958.2008.06229.x

Zaslaver, A., Bren, A., Ronen, M., Itzkovitz, S., Kikoin, I., Shavit, S., Liebermeister, W., Surette, M.G., Alon, U., 2006. A comprehensive library of fluorescent transcriptional reporters for *Escherichia coli*. Nat. Methods 3, 623–628. https://doi.org/10.1038/nmeth895

Zaslaver, A., Kaplan, S., Bren, A., Jinich, A., Mayo, A., Dekel, E., Alon, U., Itzkovitz, S., 2009. Invariant Distribution of Promoter Activities in Escherichia coli. PLOS Comput. Biol. 5, e1000545. https://doi.org/10.1371/journal.pcbi.1000545
